# Patterns of Evolutionary Diversification in a World‐Significant Hotspot of Salmonid Diversity: A Study of Landlocked *Salvelinus* Fish of the Kronotskoe‐Uzon Area, Kamchatka

**DOI:** 10.1002/ece3.74339

**Published:** 2026-09-23

**Authors:** Evgeny V. Esin, Ludmila S. Zinevich, Dmitriy A. Medvedev, Nikolai O. Melnik, Daria M. Panicheva, Grigorii N. Markevich

**Affiliations:** ^1^ Vitus Bering Kamchatka State University Petropavlovsk‐Kamchatsky Russia; ^2^ A.N. Severtsov Institute of Ecology and Evolution RAS Moscow Russia

**Keywords:** adaptive radiation, hotspot of biodiversity, microevolution, phylogeny, salmonids

## Abstract

Freshwater fish are prone to forming polymorphisms beneficial for resource partitioning and rapid adaptation. Genetically distinct ecomorphs emerge in a mosaic, forming local centers of biodiversity. The mechanisms behind this process are of particular interest, as they facilitate the study of speciation. Here we examine the patterns of evolution in the endemic charrs (genus *Salvelinus*) that populate the Kronotskoe‐Uzon drainage system. This is one of the most phenotypically diverse and complicated assemblages of salmonids on Earth. By analyzing SNPs in SLAF sequences of 107 specimens covering all local diversity and 55 specimens from nine populations of the potential ancestor, we investigated the phylogenetic relationships, the timeline of diversification, genetic structure, and historical gene flow within the population system. Our findings suggest that the endemic assemblage diverged from the widespread anadromous *Salvelinus malma* of eastern Kamchatka, which probably invaded the basin in several waves. Ten of the 14 genetically distinct ecomorphs diversified in Lake Kronotskoe at the center of the drainage system. The assemblage formed in the post‐LGM—Holocene time as a result of inter‐lake multistep adaptive radiation and specialization of hybridogenic populations, as well as the secondary expansion of endemic charrs into peripheral waterbodies. The steps of ecomorph divergence coincide well with the main geological and paleoclimatic events in the area. Although the diversity of Kronotskoe‐Uzon charrs is unique, faunal radiation has proceeded through several archetypal ecological modes implemented by freshwater fishes in dozens of reservoirs across the Holarctic. The reason for this increased diversity is the active habitat restructuring and enhanced ecological opportunities provided by the large lake–river system in the moderate climate.

## Graphical abstract

We have examined the phylogenetic relationships, genomic structure, admixture, and historical gene flow among the endemic charrs from the Kronotskoe‐Uzon water system in Kamchatka, in order to reconstruct their evolutionary history. We found that this unique diversity formed in the post‐LGM—Holocene time as a result of several waves of *Salvelinus malma* invasion, multistep adaptive radiation involving introgressive hybridization in the center of the hotspot and secondary spreading of endemic morphs into peripheral waterbodies.

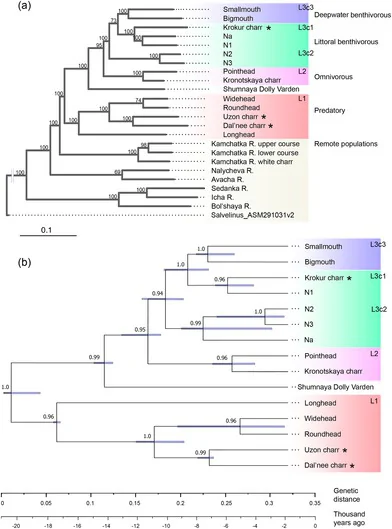

## 1 Introduction

The evolution of life is an ongoing process that has repeatedly enabled the full spectrum of flora and fauna to evolve throughout Earth's history. When ecosystems are not affected by drastic environmental changes, evolution can be described as a multi‐step process in which the most viable environmental gradients are first exploited, followed successively by secondary ones, until maximal diversity is achieved and evolution reaches a state of stasis (Simpson 1953; Birk et al. 2020; Schluter 2000). Globally, the essence of these multi‐step processes is evident in the great extinctions and subsequent recoveries of the world's fauna, as well as in episodes of deep‐ocean recolonization (Priede and Froese 2013; Baucon et al. 2023). On smaller timescales, these processes can be observed in ecosystems affected by Pleistocene–Holocene climatic fluctuations (Hewitt 2000; Lawrence and Hobeak 2000; Montgomery 2000), or in ecosystems that have originated de novo following local geological or climatic events (Whittaker et al. 2017). Such newly formed ecosystems provide an opportunity to trace the ecological and genetic fundamentals of evolution shortly after the initial stages have been completed and before stasis is reached, given their relatively long‐term stability over periods of 10^4^–10^6^ years (Simpson 1953; Stroud and Losos 2016). Consequently, investigating the fauna of these local ecosystems could help bridge the knowledge gap between microevolutionary processes and the macroevolution of global fauna, a relationship which remains poorly understood.

A paradigmatic instance of ecologically driven microevolution is evidenced by the salmonid diversification in postglacial lakes (Robinson and Parsons 2002). Numerous geographically remote adaptive diversifications of trouts, charrs, and whitefishes exhibit considerable similarity to one another (Taylor 1991; Skúlason et al. 2019; Blain et al. 2023). They consist of genetically distinct co‐occurring ecomorphs that have acquired phenotypic differences beneficial for resource partitioning and ecological specialization (Streelman and Danley 2003; Hendry 2009; Schluter and Conte 2009). The parallel and, to some degree, repeatable diversifications suggest that natural selection promotes similar phenotypes under similar genetic and developmental constraints (Brakefield 2011; Jamie and Meier 2020; Losos et al. 1998). For numerous assemblages to resemble each other, however, the ecological processes driving diversification must also be repeatable (Schluter 2000; Gillespie 2004; Yoder et al. 2010). Common gradients available to salmonids include “lake‐to‐tributary,” “pelagic‐to‐benthic,” and “coastal‐to‐deepwater” resource partitioning (Snorrason et al. 1994; Markevich and Esin 2018a). Herewith, with few exceptions, no more than four monophyletic salmonid ecomorphs diversify in local waterbodies (Salisbury and Ruzzante 2022; Blain et al. 2023). It appears that limited niche possibilities and taxon‐specific constraints on phenotypic diversification may reduce the potential for novel adaptive morphs to evolve (Kinzig et al. 1999).

The more complex salmonid diversifications are represented by charrs (genus *Salvelinus*) in the Arctic zone. Recently, the assemblage comprising six genetically differentiated ecomorphs of *Salvelinus alpinus* was discovered in the 2600‐km^2^ Tasersuaq‐Saqqaata Tasia water system in Greenland (Doenz et al. 2019). Furthermore, eight ecomorphs were found in the 3600‐km^2^ Lama‐Kapchuk water system in Taimyr (Osinov et al. 2022). In both cases, the observed diversity emerged beyond the Arctic Circle in the postglacial time due to the interaction between two closely related phylogenetic lineages, their limited hybridization, and sympatric diversification occurring simultaneously along several aforementioned archetypical environmental gradients.

In light of this evidence, the diversity of charr fauna inhabiting the adjacent Kronotskoe and Uzon basins in east Kamchatka appears to be unexpected. This relatively small water system (area ~2500 km^2^) is home to 14 endemic ecomorphs (Esin, Shkil, Shulgina, et al. 2024; Esin, Melnik, et al. 2026) derived from *Salvelinus malma* (Woronowicz et al. 2024). The assemblage comprises most of the archetypal adaptive variants presented in various combinations in other salmon diversifications. Unlike the complex *S. alpinus* diversifications in the High Arctic, which are affected by human activities, fisheries and global warming (Gladyshev 2021; Jacobsen et al. 2023), the Kronotskoe‐Uzon water system has remained intact, having been protected by a biosphere reserve since 1934. The postglacial isolation of the Kronotskoe and Uzon basins (Shantser and Melekestsev 1967) forced the rapid radiation of charrs across vacant ecological niches. Furthermore, the water system location in a temperate climate zone characterized by the earlier retreat of Pleistocene glaciers and more favorable ecological conditions has allowed charr diversification to occur across a wider range of environmental gradients over a longer period of time. Judging by geological surveys (Gorbach and Rogozin 2023), the paleo‐lake existed at the site at the end of the Last Glacial Maximum (LGM), potentially providing a refuge for charr diversification already that time. Thus, the Kronotskoe‐Uzon charr fauna is a unique model for studying evolutionary divergence in salmonids.

The hotspot consists of sympatric ecomorphs exploiting ecological opportunities of the Lake Kronotskoe basin, geographically isolated ecomorphs of small peripheral waterbodies and the anadromous ecomorph of the outflowing river (Esin, Shkil, Shulgina, et al. 2024; Esin, Melnik, et al. 2026; Esin, Zinevich, et al. 2026). The only fish species co‐occurring with charrs in Lake Kronotskoe is the resident sockeye salmon *Oncorhynchus nerka* . The ecology and morphology of Kronotskoe‐Uzon charrs have been relatively well studied (Markevich, Esin, Saltykova, et al. 2017; Markevich, Esin, Busarova, et al. 2017; Markevich et al. 2021; Esin et al. 2020; Esin, Shkil, Shulgina, et al. 2024). Analysis of microsatellite (STR) and mitochondrial DNA markers revealed genetic differentiation among all endemic ecomorphs, also indicating their complex demographic history with a mismatched population size declines (Esin et al. 2020; Esin, Melnik, et al. 2026). However, the evolutionary history of the assemblage is not entirely unclear.

In the present study, we employed genome‐wide methods attempting to resolve the complex diversification patterns of the Kronotskoe‐Uzon charrs. Our study aimed to: (i) reconstruct the phylogeny of the assemblage, (ii) date charr diversification in the context of historical geological and climatic events, (iii) evaluate the assemblage genetic structure and (iv) identify the role of historical and current hybridization in the evolutionary history of the assemblage. To address these issues, we analyzed SNPs in specific‐locus amplified fragments of the genomes of all endemic ecomorphs and surrounding Kamchatkan populations.

We hypothesized that the unique diversity of Kronotskoe‐Uzon charrs arose from several pre‐Holocene invasions of *S. malma* into the paleo‐Kronotskoe basin. This resulted in the formation of several evolutionary lineages that adopted distinct ecological specializations. During the Holocene, parallel lineage diversification occurred along archetypal ecological gradients in Lake Kronotskoe, accompanied by invasions of endemic charrs into peripheral waterbodies. The initial diversification was driven by gene flow between lineages, which decreased significantly following the emergence of morphs that were strictly adapted.

## 2 Material and Methods

### 2.1 Description of Charr Diversity

**FIGURE 1:**
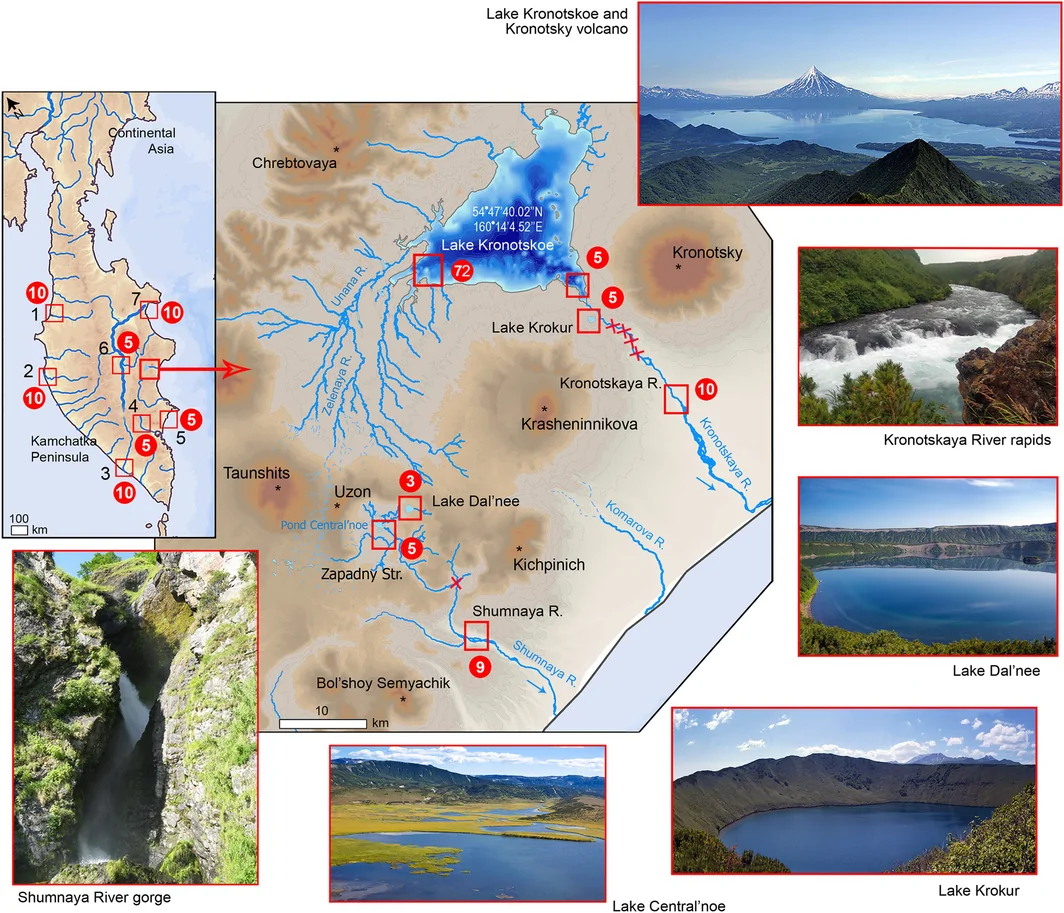
Schematic map of the Kronotskoe‐Uzon water system on the Kamchatka Peninsula. The captioned squares indicate the sampling locations of charrs, the numbers in the adjacent circles show the number of specimens used for the genome‐wide analysis. Additional sampling sites of *Salvelinus malma* are: #1—Sedanka River, #2—Icha River, #3—Bol'shaya River, #4—Avacha River, #5—Nalycheva River, #6—Kamchatka River upper course, and # 7—Kamchatka River lower course including five specimens of Dolly Varden and five specimens of the piscivorous white charr. The photos represent waterbodies of the catchment. Symbols: *, volcano peaks; x, waterfalls and rapids; …, temporary watercourses; →, flow direction.

The endemic assemblage of Kronotskoe‐Uzon charrs inhabits the large landlocked Lake Kronotskoe (surface area 246 km^2^, depth up to 136 m), its tributaries, the outflowing river, and the small waterbodies isolated on the periphery of the initial watershed due to volcanic activity (Figure 1). Ten ecomorphs with contrasting adaptive morphologies can be caught together in Lake Kronotskoe during the summer foraging period. Their strict reproductive homing (Markevich et al. 2021) prevents modern cross gene flow (Esin et al. 2020; Esin, Shkil, Shulgina, et al. 2024; Esin, Melnik, et al. 2026). Based on our previous studies of the ecological and morphological characteristics of endemic ecomorphs (Busarova et al. 2017; Markevich, Esin, Saltykova, et al. 2017; Markevich, Esin, Busarova, et al. 2017; Markevich et al. 2018; Esin, Shkil, Shulgina, et al. 2024), we categorized them into four groups: predatory, omnivorous, littoral benthivorous, and deepwater (Table 1).

**TABLE 1:** Classification of the Kronotskoe‐Uzon charrs based on morphological and ecological characteristics, sampling location and the number of specimens analyzed.

Habitats	Lineage (specialization)	Ecomorph	Feeding, lifestyle (Fork length, cm: mean mature, maximal)	External appearance	Sampling location	*N*
Lake Kronotskoe	L 3, clade 3 Deepwater benthivorous	Smallmouth	Insectivory, lacustrine hypolimnetic (20, 33 cm)	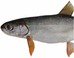	Lake Kronotskoe hypolimnion	11
		Bigmouth	Benthivory, profundal (26, 36 cm)	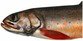	Lake Kronotskoe profundal zone	5
Lake Kronotskoe and tributaries	L 3, clade 1 Littoral benthivorous	Na (nosed)	Littoral insectivory, lacustrine‐addfluvial (32, 40 cm)	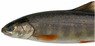	Lake Kronotskoe littoral	5
		N1 (lipped nosed)	Amphipod specialist, lacustrine‐addfluvial (33, 41 cm)	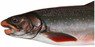	Lake Kronotskoe littoral	5
	L 3, clade 2 Littoral benthivorous	N2 (sharpnosed)	Amphipod specialist, lacustrine‐addfluvial (35, 42 cm)	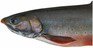	Lake Kronotskoe littoral	7
		N3 (shovelnosed)	Amphipod specialist, lake bays‐addfluvial (34, 46 cm)	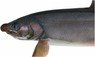	Lake Kronotskoe littoral	5
	L 1 Predatory	Longhead	Pelagic piscivory, lacustrine‐addfluvial (49, 66 cm)	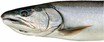	Lake Kronotskoe pelagic zone	16
		Widehead	Demersal piscivory, lacustrine‐addfluvial^a^ (37, 75 cm)	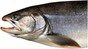	Lake Kronotskoe profundal zone	12
		Roundhead	Piscivory, fluvial (28, 41 cm)	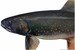	Kronotskaya River source	5
	L 2 Omnivorous	Pointhead	Inshore omnivory, lacustrine‐addfluvial (31, 42 cm)	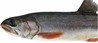	Lake Kronotskoe littoral	6
Kronotskaya River		Kronotskaya charr	Omnivory, anadromous (35, 65 cm)	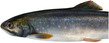	Kronotskaya River lower course	8
Uzon basin	L 1 Derivative of predatory	Uzon charr	Omnivory, lacustrine‐downfluvial^a^ (44, 55 cm)	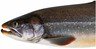	Lake Central'noe littoral	5
		Dal'nee charr	Piscivory, lacustrine (42, 48 cm)	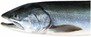	Lake Dal'nee pelagic zone	3
Lake Krokur	L 3 clade 1 Derivative of nosed	Krokur charr	Omnivory, lacustrine (19, 58 cm)	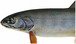	Lake Krokur pelagic zone	5
Shumnaya River		Dolly Varden	Omnivory, anadromous (39, 70 cm)	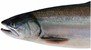	Shumnaya River lower course	9

^a^ These ecomorphs also form sedentary riverine derivatives that spawn alongside the larger migratory individuals in the tributaries of Lake Kronotskoe (Esin et al. 2015; Markevich and Esin 2018b).

Our recent analysis of the SNP‐based phylogeny revealed that the isolated ecomorphs of small peripheral waterbodies have originated from the Lake Kronotskoe endemics (Esin, Zinevich, et al. 2026). The ancestor of the predatory charr colonized two waterbodies of the Uzon caldera located to the south (Figure 1 and Table 1): the source of the Shumnaya River and Lake Dal'nee (0.8 km^2^, 50 m deep), which is a flooded maar crater (Esin et al. 2015; Esin, Zinevich, et al. 2026). The interconnection between the Lake Kronotskoe basin and the stream flowing into the Uzon caldera was blocked around 8.5 kya (Ponomareva et al. 2006; Esin, Zinevich, et al. 2026). Another isolated population from the drainless Lake Krokur (Figure 1 and Table 1) originated from the littoral benthivorous charrs (Esin and Markevich 2019; Esin, Zinevich, et al. 2026).

The anadromous charr reproduces in the Kronotskaya River downstream of the rapids. This ecomorph can be classified as a part of the Kronotskoe‐Uzon assemblage, as it experiences gene flow from the endemic ecomorphs running downstream from Lake Kronotskoe through the rapids (Esin, Melnik, et al. 2026; Esin, Zinevich, et al. 2026). The anadromous Dolly Varden, which is presumably close to the assemblage ancestor, reproduces in the rivers of the Kronotsky Bay, including the lower reaches of the Shumnaya River. No substantial gene flow with endemic charr ecomorphs, including the Kronotskaya charr, was detected for the Shumnaya River Dolly Varden (Esin, Zinevich, et al. 2026).

### 2.2 Sample Collection

We collected fin clips of the Kronotskoe‐Uzon charrs caught with gill nets with permission no. 041 dated 10.07.2023, issued by the Far Eastern Interregional Administration of Rosprirodnadzor. Endemic charrs were sampled in Lake Kronotskoe, Lake Central'noe (the Shumnaya River headwaters), Lake Dal'nee and Lake Krokur, as well as in the Kronotskaya River lower course (Figure 1). Only large adult individuals with distinct morphological features and clear identity were subsampled from the mixed catches. Anadromous Dolly Varden was caught in the Shumnaya River, as well as in six remote rivers in the east and west of Kamchatka. Samples of the resident piscivorous white charr were also taken from the Kamchatka River in order to determine its relationship with the predatory charr of Lake Kronotskoe. In accordance with the law (no. 166‐FZ), fishing for Dolly Varden outside the reserve territory does not require special permission.

In total, 107 specimens from the Kronotskoe‐Uzon water system and 55 specimens from remote rivers were used for the analysis (Figure 1 and Table 1). Putative hybrids between the anadromous ecomorph of the Kronotskaya River and the endemic ecomorphs of Lake Kronotskoe were excluded from the analysis based on preliminary tests for genetic admixture (Esin, Zinevich, et al. 2026).

In all cases, fin clips were preserved in 96% EP ethanol and high‐molecular‐weight DNA was extracted with the QIAamp DNA Mini Kit (Qiagen, Hilden, Germany) according to the manufacturer's protocol. DNA concentration was measured using the Qubit 3.0 Fluorometer and the dsDNA HS Assay Kit (Invitrogen, USA), and DNA integrity was evaluated by electrophoresis on a 1% agarose gel.

### 2.3 SLAF Sequencing

For Specific‐Locus Amplified Fragment sequencing (SLAF‐seq) library preparation, genomic DNA was digested with *RsaI* and *HaeIII* (NEB, USA). The 3′ ends of the fragments were then adenylated (A‐tailed) to facilitate adapter ligation. Dual‐indexed sequencing adapters were ligated to the A‐tailed fragments. The adapter‐ligated DNA was then PCR amplified and the PCR products were purified using VAHTS DNA Clean Beads (N411‐03, Vazyme, China). The purified PCR products were separated on a 2% agarose gel and the DNA fragments within the target size range were excised and gel‐purified. This size‐selected DNA was subjected to a second round of PCR amplification, followed by another bead‐based purification, to produce the final SLAF‐seq library. The completed libraries from 163 samples were quantified with the Qubit 3.0 fluorometer, and fragment size distribution was verified using a Qsep‐400 capillary fragment analyzer. Finally, the libraries were sequenced on an Illumina NovaSeq X Plus platform (Illumina, San Diego, CA, USA) to generate 150 bp paired‐end reads, following the manufacturer's instructions. Quality control tests were performed using FASTQC v0.11.8 (Andrews 2010) and MultiQC v1.13 (Ewels et al. 2016).

### 2.4 SNP Calling and Filtering

Paired, demultiplexed reads were filtered and adapter‐trimmed using process_ragtags (v1.44) from STACKS (v2.68; Catchen et al. 2013), ignoring the restriction sites (command ‐‐disable_rad_check). The filtered reads were mapped to the *Salvelinus* sp. genome ASM291031v2 (GeneBank accession number: GCF_002910315.2) using BWA MEM (v0.7.17‐r118; Li and Durbin 2009) with the mean success = 97.9% (parameters: ‐‐M and ‐R to define read groups). The aligned reads were sorted and indexed with SAMtools (v1.6; Li et al. 2009). Variant calling was performed using the reference‐based pipeline implemented in the ref_map.pl. script from STACKS, with ‐r 0.50 ‐‐vcf ‐‐vcf‐all for the initial run in the ‘populations’ module. We flagged putative paralogs as loci showing Ho > 0.6 and Fis < −0.5 in all groups based on per‐locus summary statistics from “populations,” and created a whitelist of the remaining loci. We then exported two filtered datasets using “populations”: a strict one (−r 0.70, one SNP per locus ‐‐write‐single‐snp, ‐‐vcf, ‐‐plink, ‐‐ordered‐export, ‐W) for PCA and admixture analysis, and a more permissive one (‐r 0.50, all SNPs retained, ‐‐fstats, ‐‐vcf, ‐‐ordered‐export, ‐W) for phylogenetic and introgression analyses.

### 2.5 Extent of Genomic Variation

In total, we obtained 2860 million paired reads across 162 samples, with a mean of 10.5 million paired reads per sample. “Gstacks” inferred genotypes using a maximum likelihood framework across 14,274,074 loci (mean length 229 bp), with 90.7% of diploid loci consistently phased. After PCR duplicate removal, the mean per sample coverage per locus was 5.4× with Std. Dev 1.6× (from 1.9× to 11.3×). Although the low coverage, “gstacks” aggregated evidence across all samples at each locus for SNP discovery and remained reliable at low individual coverage (Rochette et al. 2019). Following population‐level filtering (one sample removed; loci required to be genotyped in ≥ 50% of samples per population, −r 0.5), 5,444,965 loci were retained (mean genotyped length 206 bp), with 17,923,592 variant sites. For downstream phylogenetic analysis, we additionally filtered 1673 loci with excess heterozygosity and strongly negative Fis (putative paralogs), leaving 17,772,739 variant sites. For population structure analyses, we retained one SNP per locus using the ‐‐write‐single‐snp option in “populations,” which yielded 14,768,687 SNPs.

### 2.6 Phylogenetic Relations

Phylogenetic relations of charrs based on SNPs were analyzed using Maximum Likelihood (ML) approach in IQ‐TREE (v2.1.4; Minh et al. 2020), parameters: ‐‐m MFP for the best fit model choice, then ‐‐bb 1000 ‐alrt 1000 ‐st DNA for the fast Shimodaira‐Hasegawa likelihood‐ratio test. Multiple sequence alignment of SNPs was created using the ‐‐phylip‐var option of the STACKS module “populations,” with retention of loci genotyped in at least 80% of all samples and SNPs with a minor allele count of three (‐‐min‐mac 3). To take into account the absence of constant sites in the alignment, an ascertainment bias correction (+ASC) model (Lewis 2001) was applied to all substitution models in a best‐fit model selection process performed with model finder. The bootstrap node support was based on 1000 replicates. Heterozygous sites within each individual were encoded using IUPAC notation. The tree was rooted to the ASM291031v2 genome. As the reference genome was positioned inside one of the clades, the tree was re‐rooted using ape (v5.3; Popescu et al. 2012) in R (v3.5.0; R Core Team 2018). The resulting tree was visualized with ggplot2 (v3.4.2), ape (v5.3), phytools (v0.7.80) and ggtree (v1.14.6) in R. Monophyletic clades were collapsed for clarity.

For species tree and divergence time inference, we used the ‘snapper’ module in BEAST (v2.7; Leaché and Bouckaert 2018), that implements a Bayesian multispecies coalescent method that infers species relationships and population parameters directly from biallelic SNP data. Five samples of each ecomorph (three for the Lake Dal'nee charr) of the Kronotskoe‐Uzon assemblage were selected for the analysis. Population sizes were estimated with the coalescent‐rate parameter (initial value = 1.0, bounds 1.0 × 10^−10^–10.0) under a Gamma prior (*α* = 2.0, *β* = 1.0). The substitution model assumed fixed mutation rates (*U* = 1.0, *V* = 1.0). Likelihood settings excluded monomorphic sites, applied the coalescent‐rate pattern, and used the Beta root prior. Following Taylor et al. (2025), a strict molecular clock was applied with an initial rate of 5.97 × 10^−9^ mutations per site per generation. A lognormal prior (*M* = 1.14 × 10^7^, *S* = 0.5, mean in real space) was used then to calibrate the scale of genetic distances. As the ecomorphs are characterized by significant demographic events and periods of low effective population size (Esin, Melnik, et al. 2026; Esin, Zinevich, et al. 2026), we expected gene drift to obscure estimates of the mutation rate. To make time estimates, therefore, the Normal MRCA prior was set based on geological data for the Krokur charr clade. This waterbody formed ~6.0 kya (Ponomareva et al. 2017) and was most likely populated immediately after its formation by an ancestor of one of the endemic Lake Kronotskoe lineages (Esin, Zinevich, et al. 2026), via a stream located at the site of the maar. The Yule speciation model was used as the tree prior, with a birth‐rate parameter of 1.0 and a Gamma prior (*α* = 2.0, *β* = 15.0).

### 2.7 Genetic Structure

To summarize genetic variation into successive orthogonal projection, we ordinated samples using principal component analysis (PCA) in PLINK (v2.00a2.3LM; Chang et al. 2015). The visualization was made in R with ggplot2, ggrepel (v0.8.2) and patchwork (v1.1.1). First, PCA was performed for all samples, then for samples of the Kronotskoe‐Uzon assemblage only. Additionally, the pairwise *F*_ST_ values (Reich et al. 2009) among the Kronotskoe‐Uzon ecomorphs were calculated using “populations” of STACKS.

### 2.8 Historical Gene Flow

We used Patterson's D statistics from DSUITE (v0.4; Malinsky et al. 2021) to test for gene flow between the Kronotskoe‐Uzon ecomorphs. This method is a widely used and robust tool to detect hybridization between closely related groups and to distinguish it from incomplete lineage sorting. For this analysis, the consensus tree from IQ‐TREE was used with the “d‐trios” function to calculate D‐statistics for all possible combinations of ecomorph hybridization. To assess the significance of the D‐statistics, Z‐scores and their standard errors were calculated with the jackknife approach after adjusting for multiple comparisons (Durand et al. 2011). The number of blocks used in the jackknife procedure (‐‐JKnum parameter) to assess the significance of D was tested across a range of values from 20 to 60 to ensure consistency of results. The results were visualized as a heatmap using the dtools.py script from DSUITE.

## 3 Results

### 3.1 Phylogeny of Charrs

**FIGURE 2:**
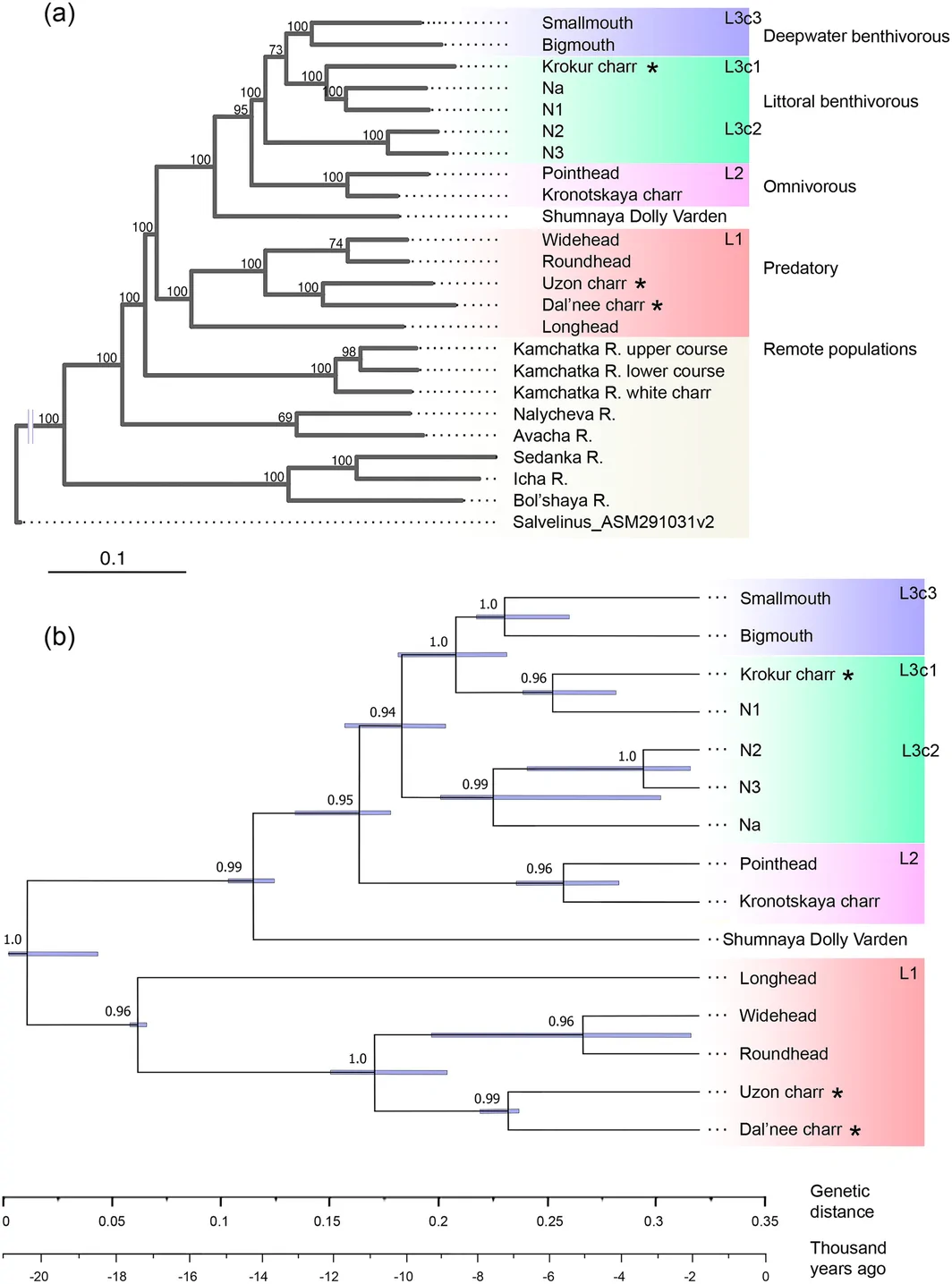
Genetic phylogeny of the Kronotskoe‐Uzon charrs based on 17,772,739 SNPs. (a) Maximum likelihood tree generated from the TVMe + R3 + ASC substitution model. The numbers represent the bootstrap support values for each node; the scale bar shows nucleotide substitutions per site. (b) Dated BEAST tree constructed using the Bayesian multispecies coalescent method. The support values (posterior probabilities) are shown above the branches. The scale bar represents the expected substitutions per site and the timeline anchored to the divergence of the Krokur morph. Node bars signify the 95% HPD age range. The ecomorphs are labeled and grouped into phylogenetic lineages (L1–4) as shown in Table 1; * indicates derivative populations of Lake Kronotskoe charrs.

The best‐fit nucleotide substitution model for resolving charr phylogeny was TVMe + R3 + ASC. The resulting tree (a log‐likelihood of the consensus tree = −541,6032.3) supported the monophyly of charrs from the Kronotsky Bay basin (Figure 2a). Of the *S. malma* populations from other Kamchatkan waterbodies, the west coast Dolly Varden exhibited the greatest degree of segregation. The Kamchatka River charrs were the closest relatives of the Kronotskoe‐Uzon assemblage. The piscivorous white charr of the Kamchatka River was found to form the clade sister to the Dolly Varden clade of the same river.

Three lineages of the Kronotskoe‐Uzon charrs could be identified on the ML tree, and these correspond to the previously allocated morpho‐ecological groups (Table 1). The initial branching of the predatory charr lineage (longhead, widehead and roundhead morphs, as well as the derived Uzon and Dal'nee charrs) was highly supported (100% bootstrap). In the second cluster, the Dolly Varden from the Shumnaya River split from the rest of the two endemic lineages (95% support). The omnivorous Kronotskaya charr and the pointhead morph formed a sister lineage relative to the lineage of the Lake Kronotskoe benthivorous morphs (100% support). The latter diverged into three clades: the central one comprises the littoral nosed charrs (Na and N1) and their derivative from Lake krokur, the second comprises the morphologically specialized N2 and N3 nosed morphs (73% support), and the third comprises the deepwater smallmouth and bigmouth morphs (100% support) (Figure 2a).

### 3.2 Timeline of Charr Diversification

Phylogenetic analysis in BEAST (Figure 2b; sum coverage 95.38%) revealed a tree topology highly similar to the ML tree. The only differences were the position of the Na morph within the third phylogenetic lineage (clade no. 1 or clade no. 2) and the ratio of certain branch lengths. The first lineage of predatory charrs was a sister to all other Kronotskoe‐Uzon charr fauna. Dolly Varden from the Shumnaya River was identified as an outgroup to the lineages no. 2 and 3.

The model auto‐correction for the divergence time (ACT) of the Krokur charr was 5925 kya, mean distance = 0.258 ± 6.05e^−3^ (st.dev. = 0.0209). Based on this time anchor, the first lineage of predatory charrs diverged from the Kronotsky Bay population system around 20 kya, with the longhead morph segregating 17 kya, that is, prior to the isolation of Lake Kronotskoe by lava. The divergence of the ancestor of nosed and deepwater charrs from Dolly Varden coincided with the time of lake isolation occurred ~14 kya. The Uzon catchment was colonized 11 kya. The clade of deepwater charrs diverged approximately 8.6 kya. The diversification of clades into the current ecomorphs took place around 7.5–3.5 kya. The pointhead morph diverged from the Kronotskaya charr ~5.8 kya.

### 3.3 Genetic Structure of Charr Assemblage

**FIGURE 3:**
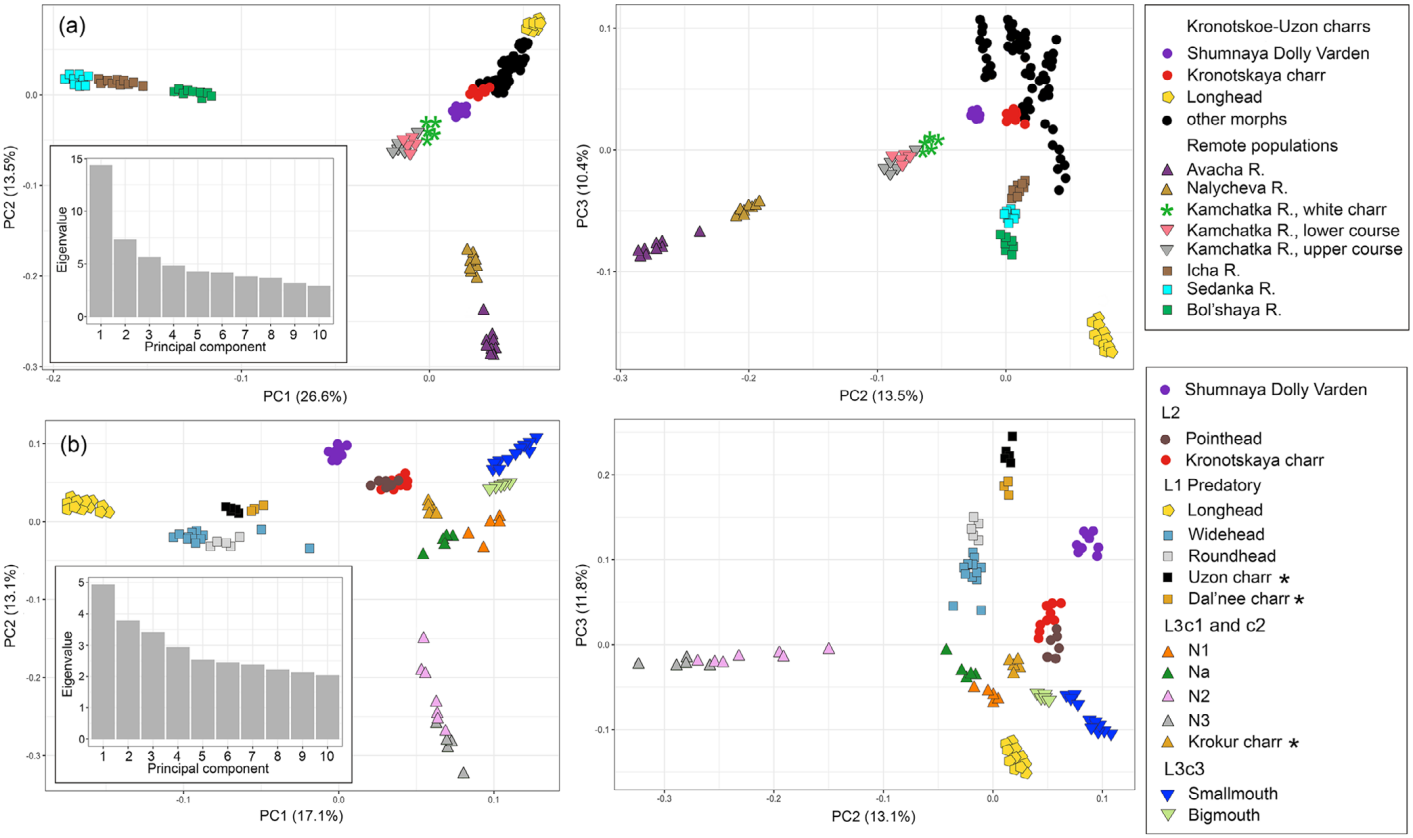
Principal component analysis of the genetic structure of the Kronotskoe‐Uzon charr based on 14,768,687 SNPs. The graphs above (a) show the results of the analysis along PC1–PC2 and PC2–PC3 for all samples, including *Salvelinus malma* from remote rivers. The graphs below (b) show the results of the analysis along PC1–PC2 and PC2–PC3 for the Kronotskoe‐Uzon charr assemblage. The amount of variation in the dataset explained along each axis is shown in parentheses. PC eigenvalues for both steps of PCA are given as histogram plots in the inserts. The charr ecomorphs are labeled and grouped into phylogenetic lineages and clades as shown in Figure 2 and Table 1; * indicates derivative populations of Lake Kronotskoe charrs.

The PCA summarized 50.5% of the genetic variation across the first three principal components, and half of this accounted for the variation between the Kronotskoe‐Uzon charrs and populations of remote rivers. West coast populations of Dolly Varden were clearly distinct from the remaining fauna and from each other along PC1 (Figure 3a). The separation of south‐eastern Kamchatkan populations was apparent along PC2. The population system of the Kamchatka River, including the white charr, formed a compact area in the PC space close to the area occupied by the Kronotskoe‐Uzon charrs. The analysis differentiated all landlocked Kronotskoe‐Uzon charrs from the Shumnaya River Dolly Varden, but did not differentiate the landlocked charrs from the Kronotskaya charr. PC3 also distinguished the longhead morph from the other Kronotskoe‐Uzon ecomorphs.

To separate the endemic Kronotskoe‐Uzon charrs, we excluded all Dolly Varden specimens from remote Kamchatkan waterbodies from the PCA. In this case, the first three principal components summarized 42% of the variation. PC1 separated the longhead morph from the rest predatory morphs, with the related Uzon charrs, the Shumnaya River Dolly Varden and all the other ecomorphs (Figure 3b). PC2 separated the N2 and N3 morphs from the others, while PC3 differentiated the Uzon and Dal'nee charrs. The deepwater smallmouth and bigmouth morphs grouped together. The pointhead morph from Lake Kronotskoe and the Kronotskaya charr were closest to each other. Overall, each ecomorph formed a compact area and could be separated in the combined PC1–PC2–PC3 space. No individuals with an intermediate position that could be interpreted as between‐lineage hybrids were found.

All *F*_ST_ pairwise comparisons of the Kronotskoe‐Uzon charrs were statistically significant, with values ranging from 0.044 (Na vs. Kronotskaya charr, *p* = 0.035) to 0.239 (bigmouth vs. Uzon charr, *p* < 0.001) (Table 2). The isolated peripheral Uzon, Dal'nee and Krokur charrs were the most distant (average *F*_ST_ ≥ 0.170). Among the sympatric Lake Kronotskoe ecomorphs, the longhead and N3 morphs were the most differentiated (average *F*_ST_ = 0.154–0.158), while the widehead and smallmouth morphs were the least differentiated (=0.100). The *F*_ST_ values were as follows: between the deepwater (Lineage 3 clade 3) and predatory (L1) charrs = 0.136, between the deepwater (L3c3) and nosed (L3c1 + c2) charrs = 0.153, between the predatory (L1) and nosed (L3c1 + c2) charrs = 0.139 (*p* < 0.001 in all cases). Estimated *F*_ST_ values between the Kronotskaya charr (L2) and the deepwater (L3c3), nosed (L3c1 + c2) and predatory (L1) charrs were 0.114, 0.063 and 0.097, respectively (*p* ≤ 0.01). The *F*_ST_ between the Kronotskaya charr and the pointhead morph (both L2) was 0.079 (*p* = 0.009).

**TABLE 2:** Pairwise *F*_ST_ values among the Kronotskoe‐Uzon charr ecomorphs based on 14,768,687 SNPs.

Ecomorph	L	W	P	R	D^a^	U^a^	K^a^	Na	N1	N2	N3	S	B
Kronotskaya charr	0.097	0.065	0.059	0.088	0.145	0.136	0.153	0.044	0.048	0.100	0.129	0.080	0.129
Longhead (L)		0.081	0.108	0.098	0.156	0.130	0.187	0.130	0.125	0.120	0.133	0.111	0.139
Widehead (W)			0.095	0.057	0.121	0.109	0.174	0.117	0.112	0.105	0.119	0.089	0.126
Pointhead (P)				0.130	0.197	0.167	0.162	0.158	0.142	0.142	0.158	0.096	0.160
Roundhead (R)					0.183	0.159	0.181	0.184	0.158	0.144	0.172	0.105	0.185
Dal'nee charr (D)^a^						0.098	0.213	0.172	0.169	0.166	0.178	0.143	0.197
Uzon charr (U)^a^							0.240	0.245	0.202	0.185	0.225	0.125	0.239
Krokur charr (K)^a^								0.121	0.146	0.184	0.226	0.161	0.229
Nosed Na									0.118	0.167	0.213	0.098	0.184
Nosed N1										0.149	0.181	0.088	0.168
Nosed N2											0.082	0.101	0.162
Nosed N3												0.113	0.202
Smallmouth (S)													0.103

*Note:* *F*_ST_ values: 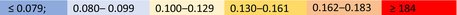. Charr ecomorph labels are listed in Table 1.

^a^ Indicates derivative populations of Lake Kronotskoe charrs.

### 3.4 Historical Gene Flow

**FIGURE 4:**
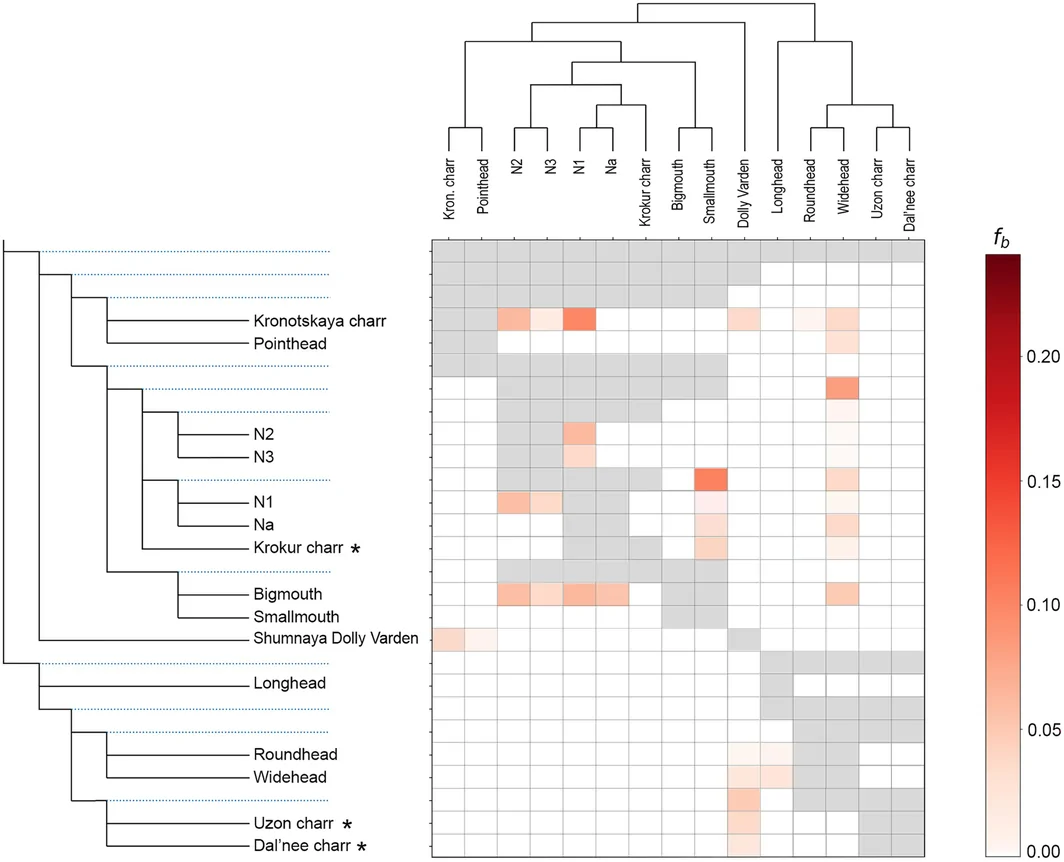
Matrix of inferred introgression proportions as estimated from gene trees for the Kronotskoe‐Uzon charrs employing the set of 17,772,739 SNPs. The matrix shows the inferred f‐branch metric, reflecting excess allele sharing between the branch of the ‘laddered’ full‐phylogenetic tree on the *Y*‐axis (relative to its sister branch) and the branches defined on the *X*‐axis. The dotted lines of the full‐phylogenetic tree represent inferred ancestral genotypes. Gray cells indicate comparisons that cannot be made owing to tree topology; white cells represent no evidence of gene flow. The charr ecomorphs are labeled as shown in Table 1; * indicates derivative populations of Lake Kronotskoe charrs.

Across 14 endemic ecomorphs and Dolly Varden from the Shumnaya River, 405 trio combinations were evaluated using D statistics, and 291 of these tests were significant after adjusting for multiple comparisons. Using the F‐branch procedure, 36 introgression events were detected, 11 of which had estimated admixture levels of over 5% (Figure 4, *f*_b_ > 0.05).

Three of the historical introgression events involved gene flow between the ancestors of the current ecomorphs. These were hybridization between the ancestors of the widehead (L1) and nosed (L3c1 + c2) morphs, between the ancestors of the smallmouth (L3c3) and Na–N1–Krokur (L3c1) morphs, and between the ancestors of the Dolly Varden from the Shumnaya River and Uzon charrs. Substantial gene flow was detected from the nosed morphs to the Kronotskaya charr (no upstream migration for the Kronotskaya charr is supposed), and from the widehead and all nosed morphs to the bigmouth morph. No signals of historical gene flow were detected in the longhead morph.

## 4 Discussion

Our data suggest that the diversity of charrs in the Kronotskoe‐Uzon water system is among the most complex for salmonids not only in north‐east Asia, but globally. Ten sympatric ecomorphs occupy the Lake Kronotskoe basin; at least three derived ecomorphs have evolved in geographically isolated peripheral waterbodies; one ecomorph of hybridogenic origin inhabits the lower course of the outflowing river. The independence of 14 faunal elements is supported by significant genetic differentiation based on genomic SNPs, as well as neutral STR markers (Esin et al. 2020; Esin, Shkil, Shulgina, et al. 2024; Esin, Melnik, et al. 2026). These faunal elements differ from each other in functional morphology (Markevich et al. 2018), trophic‐ecological markers (Esin et al. 2020; Esin, Shkil, Shulgina, et al. 2024) and preferred sites for reproduction (Esin et al. 2021; Markevich et al. 2021). They are characterized by distinct demographic histories (Esin, Melnik, et al. 2026; Esin, Zinevich, et al. 2026). Thus, the 14 endemic charr ecomorphs could be defined as independent Evolutionarily Significant Units *sensu* Crandall et al. (2000) and as the independent conservation units for the biodiversity conservation strategy in Kamchatka Krai.

The endemic assemblage diverged from the widespread migratory *S. malma* in the post‐LGM time. Our phylogenetic analysis testifies in favor of the origin of the endemic Kronotskoe‐Uzon charrs from the Dolly Varden of the Kronotsky Bay basin. As the glaciers in Kamchatka began to melt, this species gained the opportunity to reproduce in the inland waterbodies of the Kronotskoe‐Uzon area. The modern genetic differences among all ecomorphs of the Kronotskoe‐Uzon assemblage are smaller than those between populations from remote areas of the peninsula. This is evident in the SNP‐based division of geographically distant populations on the ML tree and in the principal component space. In particular, our genomic data clearly demonstrate the independent origin of the widehead morph from Lake Kronotskoe and the ecologically similar white charr from the Kronotskaya River. Genetic distances were estimated to be, on average, 0.2%–0.3% substitutions among the ecomorphs and 0.4%–0.5% among charrs of different bays of the Kamchatka coast (Esin, Zinevich, et al. 2026).

Strict monophyly was rejected for the Kronotskoe‐Uzon assemblage, as the phylogenetic tree topology indicates that the coastal population repeatedly invaded the inland basin, forming new lineages sequentially. The diversity of modern ecomorphs is the result of secondary diversification within three lineages. Despite shallow genetic differences compared to the overall genetic variation of the Kamchatkan *S. malma* , the Kronotskoe‐Uzon ecomorphs demonstrate extraordinary phenotypic diversity. This is a marker of adaptive radiation (McGee et al. 2020): a rapid increase in the number of closely related Evolutionarily Significant Units sharing the ecological opportunities of an ecosystem. Notable examples of postglacial adaptive radiation in fish include the cichlid fauna of Lake Victoria (Seehausen 2006), the Ethiopian labeobarbs (Sibbing et al. 1998; Levin et al. 2020, 2024), and the sticklebacks of British Columbia (McPhail 1992).

The radiation of Kronotskoe‐Uzon charrs has progressed in several steps. Based on the same topology of the ML and Bayesian trees, as well as the results of principal component scaling, it can be hypothesized that one of the earliest invasions into inland waters of the Kronotskoe‐Uzon area resulted in the formation of the most genetically distinct lineage that specialized into predatory charrs. The first diversification within this lineage gave rise to the genetically isolated longhead charr. Thus, this epilimnetic piscivorous morph is the oldest and most distinct member of the assemblage. This evolutionary situation distinguishes the Kronotskoe‐Uzon assemblage from other deeply diversified sympatric assemblages of salmonids, in which the deepwater morphs/species are the most specialized relic groups (Susnik et al. 2006; Osinov et al. 2022; Esin, Shkil, Zlenko, et al. 2024). While the longhead charr remained endemic to Lake Kronotskoe, the ancestor of the second clade of predatory charrs colonized the waterbodies in the Uzon caldera, giving rise to the Uzon and Lake Dal'nee charrs.

In the pre‐Holocene time, the facultatively anadromous Dolly Varden reproduced in the waterbodies of the coastal zone. This population could also exploit the benthivorous niche of paleo Lake Kronotskoe, forming a benthivorous group that foraged in sympatry with piscivorous specialists. Similar predator–benthophage pairs of *S. malma* have been observed in other lake–river systems of Kamchatka (Esin 2025). Following the isolation of Lake Kronotskoe by lava, the benthivorous charrs became separated from the Dolly Varden of the outflowing rivers. The development of lake resources and remote spawning sites led to the divergence of the resident benthivorous charrs into three clades of littoral and deepwater inhabitants, followed by secondary diversification into strictly adapted morphs within each clade. A functional analysis of conserved genome regions (Woronowicz et al. 2024) revealed that the rapid molecular diversification of charrs that lost access to the sea was driven by the relaxation of selection pressures on genes involved in smolification. Nowadays, the Dolly Varden reproducing downstream of the waterfall in the Shumnaya River forms a sister clade relative to the endemic benthivorous charrs.

The lineage of two omnivorous morphs is of particular interest in the evolutionary history of the Kronotskoe‐Uzon charrs. This lineage occupies a central position within the SNP‐based principal component space of the assemblage. We identified traces of gene flow from the nosed and widehead morphs to the omnivorous ecomorph that reproduces downstream of the lava rapids in the Kronotskaya River. Based on *F*_ST_ estimates, the Kronotskaya charr is comparatively poorly differentiated from the endemic ecomorphs. Recent analysis of genetic admixture also indicated ongoing hybridization between the Kronotskaya charr and migrants of the nosed morphs (Esin, Zinevich, et al. 2026). Unexpectedly, all SNP analyses indicate that the pointhead morph of Lake Kronotskoe is closely related to the Kronotskaya charr. Previously, it was erroneously identified as a representative of the predatory charr lineage on the basis of morphological and ecological data (Esin, Shkil, Shulgina, et al. 2024). The pointhead morph may represent a relic of one of the initially landlocked lineages, but this does not explain its genetic proximity to the Kronotskaya charr. This morph reproduces in the tributaries of the farthest lake bays (Esin, Shkil, Shulgina, et al. 2024), so its mass migration and hybridization with the Kronotskaya charr are highly unlikely. We favor the scenario in which the new pointhead morph evolved from a hybrid Kronotskaya charr that once again entered the lake via the rapids under conditions of a temporary drop of flow rate.

The Kronotskaya charr exhibit the greatest genetic diversity and heterozygosity of all the Kronotskoe‐Uzon ecomorphs (Esin, Melnik, et al. 2026; Esin, Zinevich, et al. 2026). We hypothesize that this population is enriched with specific alleles of endemic ecomorphs, which could form a genetic substrate for rapid adaptation in case of environmental changes or invasion into a new habitat. We suppose that this gene “transporter” could facilitate *S. malma* , like other ecologically plastic fish species, to rapidly produce new adaptive phenotypes (Levin et al. 2024; Esin 2025). For instance, the multiple origins of the resident phenotype in sticklebacks could be attributed to similar processes (Schluter and Conte 2009). The proposed origin of the pointhead morph resembles the secondary invasion of the haplochromine cichlid from the Nile basin into Lake Tanganyika, resulting in the formation of a new species (Meyer et al. 2015).

Our estimates of diversification times, derived from Bayesian computations, suggest a higher mutation rate in the Kronotskoe‐Uzon charrs than traditional calculations of lower vertebrate evolutionary rates imply (Feng et al. 2017; Bergeron et al. 2023). This discrepancy can be attributed to the complex demographic history of the endemic ecomorphs. Previous analyses of STR markers have revealed multiple bottlenecks and periods of low effective population size in the morphs' history (Esin, Melnik, et al. 2026). Herewith, the presence of a local Krokur isolate provides an opportunity to calibrate the diversification timeline with some assumptions. These time estimates coincide well with those of geological and paleoclimatic events in the area.

The ancestor of the endemic benthivorous charrs became isolated in Lake Kronotskoe around 14 kya, when the Kronotskaya River valley was flooded by lava. The predatory charr colonized the Uzon caldera around 11 kya via the temporary water network from the Zelenaya River headwaters (Figure 1) during the melting of the Pleistocene glaciers, which is consistent with our previous hypothesis (Esin, Zinevich, et al. 2026). The radiation of benthivorous charrs began with the onset of the Holocene, accompanied by an increase in habitat diversity and productivity in Kamchatka's lakes (Brooks et al. 2015; Kaufman et al. 2020). Around 8.6 kya, during the peak of climate warming (Brooks et al. 2015), the descendants of the benthivorous Dolly Varden split into the clades of littoral and deepwater charrs. The diversification of the clades into the current morphs was probably triggered by mid‐Holocene climate change, leading to the emergence of new spawning tributaries and the structuring of littoral habitats. Around 5 kya, the Kronotskaya charr was able to overcome the rapids and enter the lake, forming the pointhead morph. This event may have occurred during a period of reduced precipitation in Kamchatka (Brooks et al. 2015), which led to a decrease in the Kronotskaya River runoff and a temporary drop of flow rate.

Sympatric ecomorphs of radiating fish have been shown to emerge in just a few dozen generations (Hendry et al. 2000; Komarova et al. 2021). The radiation of the Kronotskoe‐Uzon charr fauna is ongoing, with several ecomorphs continuing to evolve and adapt. The most recent diversification event occurred 2–4 kya, led to the emergence of the N2 and N3 nosed morphs. In the SNP‐based principal component space, the N2 and N3 fall into the same sector as the other benthivorous ecomorphs, but stand out against the background of comparable differences among the other nosed and deepwater ecomorphs. The N2 and N3 morphs exemplify the “extralimital specialization” *sensu* Myers (1960) within the assemblage. They differ in a shortened lower jaw, enabling them to grasp crustaceans using the teeth on the vomer and the anterior edge of the dentary like tweezers (Markevich, Esin, Busarova, et al. 2017). Furthermore, the N3 morph has a hypertrophied, shovel‐shaped snout that is covered in sensitive epithelium (Kapitanova et al. 2019).

With the exception of the N2–N3 clade, we can argue that the radiation of the endemic assemblage has realized via several archetypal ecological gradients accessible to freshwater fish in dozens of reservoirs across the Holarctic. These include uneven resource use in the tributary—lake continuum due to differential migratory activity (migratory littoral—sedentary deepwater benthophages; migratory widehead—riverine roundhead morphs), utilization of pelagic versus benthic resources (longhead—widehead morphs), diversification along the depth gradient (littoral—slope—profundal ecomorphs) and secondary diversification in coastal—offshore environments (Na—smallmouth insectivores). Prey diversification has also been shown to provide a niche space for predators to radiate (Brodersen et al. 2015, 2018). The Lake Kronotskoe charrs have co‐evolved alongside their prey, forming a pelagic engulfer of planktivorous sockeye (longhead), a demersal hunter of benthivorous sockeye (widehead). Whitefishes, trouts, and some charr species have repeatedly diversified along these environmental gradients, forming ecomorphs that exhibit adaptive phenotypic traits similar to those of distinct representatives of the Kronotskoe‐Uzon fauna (Alekseyev et al. 2009; Markevich and Esin 2018a; Skúlason et al. 2019; Blain et al. 2023). Therefore, the Kronotskoe‐Uzon assemblage is a set of typical adaptive morphotypes, and its distinctive feature is the maximum variety of variants that have arisen in one water system.

Contact between different phylogenetic lineages and historical hybridization have been highlighted as important triggers of lacustrine‐riverine fish radiations (Meier et al. 2017; Levin et al. 2020). In the Kronotskoe‐Uzon charrs, historical hybridization has likely also played a role in increasing phenotypic plasticity and creating opportunities for adaptive radiation. For example, D statistics indicate the initial contact of the predatory charrs that invaded the Uzon caldera with the anadromous Dolly Varden probably occurred in the Shumnaya River headwaters prior to the formation of the waterfall.

We found clear signs of introgression between the ancestor of predatory morphs and the lineage leading to all nosed morphs. This introgression signal is not the strongest identified within the assemblage, although the predatory widehead morph migrates through the breeding grounds of the nosed morphs to spawn, potentially getting the opportunity for annual hybridization with them (Markevich et al. 2021). This confirms the effectiveness of the ecological mechanisms previously discovered for isolating morphs with riverine spawning (Esin et al. 2021; Markevich et al. 2021). The admixture of the ancestor of nosed charrs with the smallmouth morph can be explained by the accidental spawning of nosed charrs in the lake instead of the tributaries. A strong gene flow signal was detected from the widehead and nosed morphs to the deepwater bigmouth morph. Previous analyses of SNPs in conserved genome regions (Woronowicz et al. 2024) have also revealed evidence of historical hybridization between the nosed and bigmouth morphs. Notably, the bigmouth morph exhibits a morphotype characteristic of deepwater salmonids and shares few similarities with the morphotype of littoral nosed charrs (Markevich et al. 2018). This could confirm the effectiveness of natural selection in the charr radiation. Judging by the significant differentiation of all endemic ecomorphs, as determined by *F*_ST_ calculations, as well as analysis of modern genetic admixture (Esin, Melnik, et al. 2026; Esin, Zinevich, et al. 2026), the stage of active hybridization between the ecomorphs of the Kronotskoe‐Uzon assemblage has passed.

## 5 Conclusion

We can conclude that the Kronotskoe‐Uzon fauna comprises three phylogenetic lineages of *S. malma* , which representatives exhibit contrast morpho‐ecological specializations. These endemic lineages have originated from several invasions of the coastal population into the mountain drainage system. The evolutionary diversification of endemic charrs commenced in the Pleistocene, following the end of the Ice Age, and continued in several steps in the Holocene. The current diversity is complemented by the ecomorphs of hybrid origin, although hybridization is limited under modern conditions. The ecomorphs from small peripheral waterbodies within the drainage system have originated from endemic lineages rather than from external *S. malma* populations. The differentiation of all endemic Kronotskoe‐Uzon ecomorphs is possible based on the analysis of SNPs. As in many other post‐LGM fish radiations, the assemblage's range of genetic polymorphism does not correspond to its extraordinary range of phenotypic diversity.

A multitude of factors can be engaged for the explanation of the revealed diversity. These include the specific ontogenetic plasticity and ecological versatility of *S. malma* (Klemetsen 2013); an initially large population size and a rich genetic substrate for natural selection to operate; the isolation from external gene flow, etc. Although basin size (area/lake depth) was considered to be one of the main predictors of fish assemblage diversity (Recknagel et al. 2017; Doenz et al. 2019; Blain et al. 2023), the Kronotskoe‐Uzon water system is relatively small. Herewith, the climatic seasonality and nutrition abundance were shown to exert a positive effect on the complexity of *Salvelinus* assemblages (Blain et al. 2023; Tiddy et al. 2024). The lakes of the Kronotskoe‐Uzon area are oligotrophic with the signs of mesotrophy (Anisimova et al. 2025). The evidence presented supports the assumption that the enhanced ecological opportunities provided by a structurally and productively diverse ecosystem in a moderate, non‐Arctic climate are the primary reason for the high charr diversity in the Kronotskoe‐Uzon catchment. The variety of habitats and food sources, year‐round access to tributaries, as well as temperature gradients and the restructuring of the littoral in summer and winter have all played a crucial role in the adaptive radiation of *S. malma* .

## Author Contributions

**Evgeny V. Esin:** conceptualization (lead), data curation (lead), formal analysis (equal), funding acquisition (equal), investigation (lead), methodology (equal), project administration (lead), resources (lead), software (supporting), supervision (lead), validation (lead), visualization (lead), writing – original draft (lead), writing – review and editing (lead). **Ludmila S. Zinevich:** formal analysis (equal), investigation (equal), software (equal), supervision (equal). **Dmitriy A. Medvedev:** data curation (supporting), formal analysis (supporting), investigation (equal), software (supporting). **Nikolai O. Melnik:** investigation (supporting), visualization (supporting). **Daria M. Panicheva:** conceptualization (supporting), funding acquisition (lead), project administration (lead). **Grigorii N. Markevich:** conceptualization (equal), data curation (supporting), investigation (equal), writing – original draft (supporting), writing – review and editing (supporting).

## Funding

The work was carried out for the Interagency Complex Programme for Scientific Research of the Kamchatka Peninsula and Certain Water Areas in 2024–2026 (FZSS‐2024‐0002).

## Ethics Statement

All procedures with fish were carried out according to the guidelines and following the laws and ethics of the Russian Federation, and approved by the ethics committee of the A.N. Severtsov Institute of Ecology and Evolution, Russian Academy of Sciences (Approval ID N 88 issued 22.03.2024).

## Conflicts of Interest

The authors declare no conflicts of interest.

## Data Availability

Raw Illumina reads have been submitted to the European Genome‐Phenome Archive (ENA) and are available under BioProject study number ERP185516 (samples ERS28138360 … ERS28384601). https://www.ebi.ac.uk/ena/browser/view/PRJEB104211.
